# Crystal structure and characterization of the sul­fa­methazine–pi­peri­dine salt

**DOI:** 10.1107/S2053229622012050

**Published:** 2022-02-27

**Authors:** Juan Saulo González-González, Salvador Pérez-Espinoza, Francisco Javier Martínez-Martínez, Armando Pineda-Contreras, Miguel Ángel Canseco-Martínez, Marcos Flores-Alamo, Héctor García-Ortega

**Affiliations:** a Instituto de Farmacobiología, Universidad de la Cañada, Carretera Teotitlán-San Antonio Nanahuatipán, km 1.7 s/n, Teotitlán de Flores Magón, Oaxaca, 68540, Mexico; b Facultad de Ciencias Químicas, Universidad de Colima, km 9 Carretera Colima-Coquimatlán, Coquimatlán, Colima, 28400, Mexico; c Instituto de Investigación en Materiales, Universidad Nacional Autónoma de México, Ciudad de México, 04510, Mexico; d Facultad de Química, Universidad Nacional Autónoma de México, 04510, Ciudad de México, Mexico; University of Strathclyde, United Kingdom

**Keywords:** sul­fa­methazine, pi­peri­dine, IR spectroscopy, crystal structure, solid-state ^13^C NMR, thermal analysis, proton transfer, solvent-assisted grinding

## Abstract

Sulfamethazine [*N* ^1^-(4,6-di­methyl­pyrimidin-2-yl)sulfanilamide] is an anti­micro­bial drug that possesses functional groups capable of acting as hydro­gen-bond donors and acceptors, which make it a suitable supra­molecular building block for the formation of co­crystals and salts. We report here the crystal structure and solid-state characterization of the 1:1 salt pi­peri­dinium sul­fa­methazinate (PPD^+^·SUL^−^, C_5_H_12_N^+^·C_12_H_13_N_4_O_2_S^−^) (**I**). The salt was obtained by the solvent-assisted grinding method and was characterized by IR spectroscopy, powder X-ray diffraction, solid-state ^13^C NMR spectroscopy and thermal analysis [differential scanning calorimetry (DSC) and thermogravimetric analysis (TGA)]. Salt **I** crystallized in the monoclinic space group *P*2_1_/*n* and showed a 1:1 stoichiometry revealing proton transfer from SUL to PPD to form salt **I**. The PPD^+^ and SUL^−^ ions are connected by N—H^+^⋯O and N—H^+^⋯N inter­actions. The self-assembly of SUL^−^ anions displays the amine–sulfa *C*(8) motif. The supra­molecular architecture of salt **I** revealed the formation of inter­connected supra­molecular sheets.

## Abstract 2

The pi­peri­dinium–sul­fa­methazinate salt was prepared by the solvent-assisted grinding method. The ions are connected by N—H^+^⋯O and N—H^+^⋯N inter­actions. The self-assembly of sul­fa­methazinate anions displays the amine–sulfa *C*(8) motif. The supra­molecular architecture of the salt revealed the formation of inter­connected supra­molecular sheets.

## Introduction

Sulfonamides are anti­microbial drugs used for the treatment of human and veterinary bacterial infections, and act by inhibiting the enzyme di­hydro­pteroate synthase, a key enzyme involved in folate synthesis (Ovung & Bhattacharyya, 2021 ▸). The chemical structure of sulfonamides includes SO_2_, NH and NH_2_ groups capable of acting as hydro­gen-bond donors and acceptors, and also arene rings capable of forming π-inter­actions, which make them suitable supra­molecular building blocks for use in crystal engineering for the formation of pharmaceutical co­crystals (Caira, 2007 ▸).

Pharmaceutical co­crystals are crystalline materials com­posed of an active pharmaceutical ingredient (API) and a co­crystal coformer which remain together in the crystalline lattice principally *via* hydro­gen-bond inter­actions. Pharmaceutical co­crystallization offers the possibility of obtaining new solid forms of APIs and improving poor physicochemical properties (Bolla & Nangia, 2016 ▸).

Sulfamethazine (SUL) co­crystals, solvates and salts have been prepared to study its ability to form noncovalent inter­actions, amidine–imidine tautomerism and proton transfer (Ghosh *et al.*, 2011 ▸; Zhang *et al.*, 2017 ▸; Singh & Baruah, 2019 ▸). Concerning the improvement of physicochemical and pharmaceutical properties, co­crystals of sul­fa­methazine with 4-amino­salicylic acid and 4-amino­benzoic acid enhance solubility, dissolution and anti­bacterial activity (Pan *et al.*, 2019 ▸; Serrano *et al.*, 2016 ▸).

**Figure 1:**
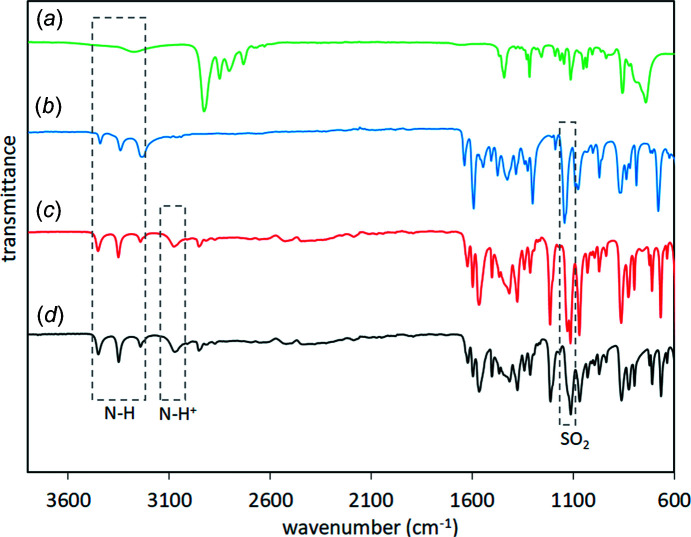
IR spectra of (*a*) PPD, (*b*) SUL, (*c*) the polycrystalline powder of **I** and (*d*) crystal **I**.

Piperidine (PPD) is a heterocyclic amine that possesses an N—H group able to act as a hydro­gen-bond donor. Combination with pharmaceutical ingredients gives rise to the formation of co­crystals (with curcumin; Sanphui & Bolla, 2018 ▸) or salts [with diclofenac (Fini *et al.* 2012 ▸) and sulfa­pyridine (Pratt *et al.*, 2011 ▸)]. The formation of co­crystals or 
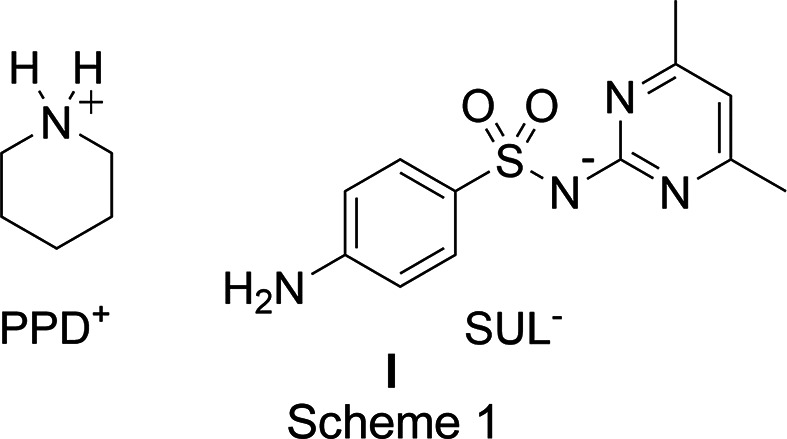
 salts can be predicted (not exactly) using the Δp*K_a_* criteria from [p*K_a_* (base) – p*K_a_* (acid)]. When the value of Δp*K_a_* is greater than 3, salt formation occurs, and when the value of Δp*K_a_* is less than 0, co­crystal formation occurs. Δp*K_a_* values between 0 and 3 do not give clear information about the co­crystal/salt preference (Kumar & Nanda, 2018 ▸). We report here a crystallization study between sul­fa­methazine (p*K_a_* = 7.40; Zhang *et al.*, 2016 ▸) and pi­peri­dine (p*K_a_* = 11.10; Luna *et al.*, 2016 ▸) (Fig. 1 ▸), producing the PPD^+^·SUL^−^ salt, **I** (Δp*K_a_* = 3.7) (Scheme 1) by solvent-assisted grinding and solvent evaporation. The solid-state characterization was performed by IR spectroscopy (IR), powder X-ray diffraction (PXRD), solid-state nuclear magnetic resonance (^13^C NMR) spectroscopy, differential scanning calorimetry (DSC) and thermogravimetric analysis (TGA). The crystal structure was ob­tained by single-crystal X-ray diffraction.

## Experimental

### Synthesis and crystallization

Sulfamethazine and pi­peri­dine were purchased from Aldrich. Di­chloro­methane and ethanol were purchased from Química Mayer. All reagents were used as received.

Sulfamethazine (0.3 g, 1.077 mmol) and pi­peri­dine (0.106 ml, 1.077 mmol), in a 1:1 molar ratio, were placed in a mortar. Before grinding, 0.5 ml of di­chloro­methane was added. The mixture was then ground with a pestle for 3 min. After the grinding time, the di­chloro­methane was evaporated and the powder was collected in the centre of the mortar. The cycle of adding 0.5 ml of di­chloro­methane and grinding for 3 min was repeated three more times until a grinding time of 12 min was reached. The polycrystalline ground powder of **I** was collected and stored in a glass vial. Single crystals suitable for X-ray diffraction were obtained from a solution of **I** in ethanol left to evaporate at room tem­per­ature.

### IR spectroscopy

The IR spectra of solid powders of SUL and PPD, the polycrystalline powder of **I** and the single crystal of **I** were acquired in a Bruker Tensor 27 spectrophotometer equipped with an attenuated total reflectance (ATR) system accessory (16 scans, spectral range 600–4000 cm^−1^, resolution 4 cm^−1^).

### Powder X-ray diffraction

Powder X-ray diffraction (PXRD) patterns of SUL, PPD and the polycrystalline powder of **I** were recorded on a PANalytical X’Pert PRO diffractometer with Cu *K*α_1_ radiation (λ = 1.5405 Å, 45 kV, 40 mA) from 2.02 to 49.93° in 2θ.

### Solid-state ^13^C NMR

Cross-polarization/magic angle spinning (CP/MAS) ^13^C NMR experiments of the solid powders of SUL and the polycrystalline powder of **I** were performed on a Bruker 400 Avance III (^13^C, 100 MHz) instrument at 25 °C. 4 mm bullet-type Kel-F zirconia rotors were used (containing about 100 mg of the sample). The spinning rate and acquisition time were 8 kHz and 32 ms, respectively. The recycle time of the pulse was 3 s. The adamantane signal was used as the external reference (δ = 38.48 ppm).

### Thermal analysis

Differential scanning calorimetry (DSC) measurements were obtained on a TA Instruments Q100 instrument. Samples placed in aluminium pans were heated from 25 to 255 °C under a nitro­gen atmosphere at a rate of 10 °C min^−1^. Thermogravimetric analysis (TGA) was performed on a TA Instruments SDT Q600 instrument. Samples placed in aluminium pans were heated from 25 to 315 °C under a nitro­gen atmosphere at a rate of 10 °C min^−1^. Heating of **I** was performed in a VelaQuin 9053A oven at 185 °C for 1 h (arbitrary time).

### Refinement

Crystal data, data collection and structure refinement details are summarized in Table 1 ▸. The H atoms of amine N—H groups were located in a difference map and refined isotropically with *U*_iso_(H) = 1.2*U*_eq_(N). H atoms attached to C atoms were placed in geometrically idealized positions and refined as riding on their parent atoms, with C—H = 0.95–0.99 Å and *U*_iso_(H) = 1.2*U*_eq_(C) for aromatic and methyl­ene groups, and 1.5*U*_eq_(C) for methyl groups.

## Results and discussion

### Solid-state characterization of salt I

The formation of the new solid phase of salt **I** was evidenced by IR spectroscopy because the IR spectrum was different from those of the starting materials PPD and SUL, showing shifts in the N—H and SO_2_ bands (Fig. 1 ▸), indicating the formation of inter­molecular inter­actions [the IR spectra of SUL and PPD were assigned according with Yang *et al.* (2005 ▸) and Güllüoğlu *et al.* (2007 ▸), respectively]. The distinctive bands in the IR spectra of the polycrystalline powder of **I** and the single crystal of **I** are those at 3076 and 3073 cm^−1^, respectively, belonging to the pi­peri­dinium N—H^+^ group (Silverstein *et al.*, 1991 ▸); also, the SO_2_ band at 1114 cm^−1^ in the polycrystalline powder and the single crystal of **I**, which is shifted to a lower wavenumber with respect to the starting material SUL (1145 cm^−1^), indicated deprotonation of the sulfonamide group, as observed in the formation of the benzamidinium sulfamerazinate salt (Kamali *et al.*, 2015 ▸) and in the formation of metallic complexes of SUL with silver and copper (Tailor & Patel, 2015 ▸). The N—H bands of SUL were also shifted from 3441 and 3339 to 3452 and 3351 cm^−1^ in the polycrystalline powder of **I**, and to 3451 and 3350 cm^−1^ in the single crystal of **I** (Fig. 1 ▸).

**Figure 2:**
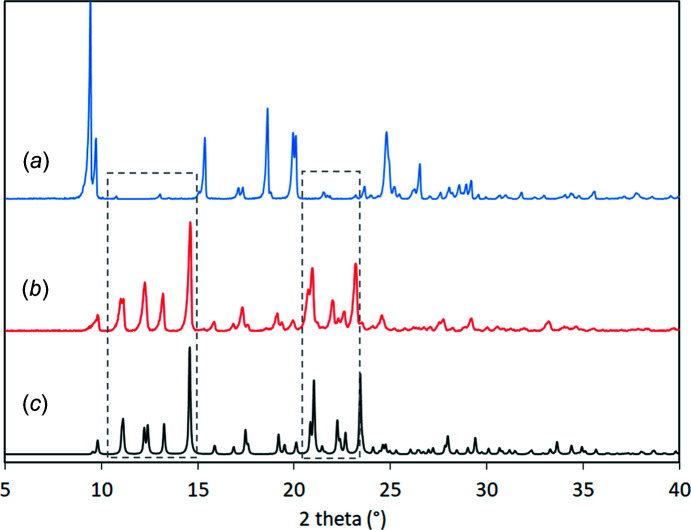
Powder X-ray diffractograms of (*a*) SUL, (*b*) the polycrystalline powder of **I** and (*c*) the simulated pattern for **I**.

The experimental PXRD pattern of SUL matched well with the simulated pattern obtained from *Mercury* (Macrae *et al.*, 2020 ▸) for the Cambridge Structural Database (CSD; Groom *et al.*, 2016 ▸) refcode SLFNMD01 (Basak *et al.*, 1983 ▸). The PXRD pattern of the polycrystalline powder of **I** was different from the PXRD pattern of SUL and matched well with the simulated pattern of crystal **I** obtained from *Mercury* (Fig. 2 ▸). The complete transformation of the starting components into the new solid phase of **I** was evidenced by the absence of the diffraction peaks at 2θ = 9.4, 15.3, 18.6, 24.7 and 26.5° belonging to the starting material SUL in the PXRD pattern of the polycrystalline powder of **I**, and the appearance of new diffraction peaks at 2θ = 11.1, 12.2, 13.2, 14.5, 20.9, 22.0 and 23.1°.

**Figure 3:**
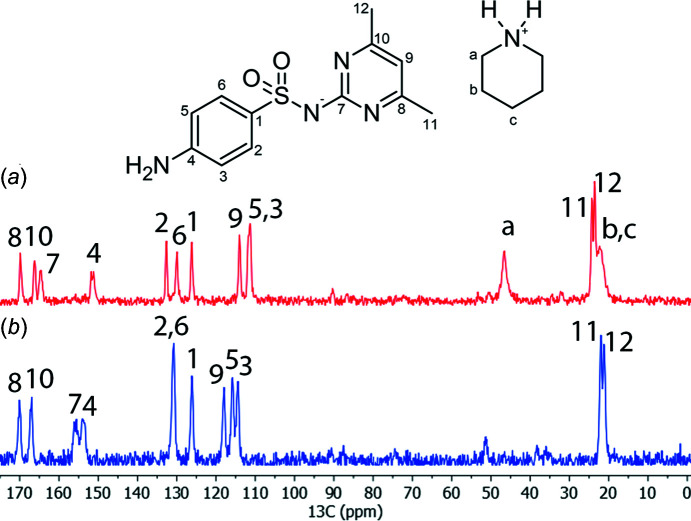
The solid-state ^13^C NMR spectra of (*a*) the polycrystalline powder of **I** and (*b*) SUL.

**Figure 4:**
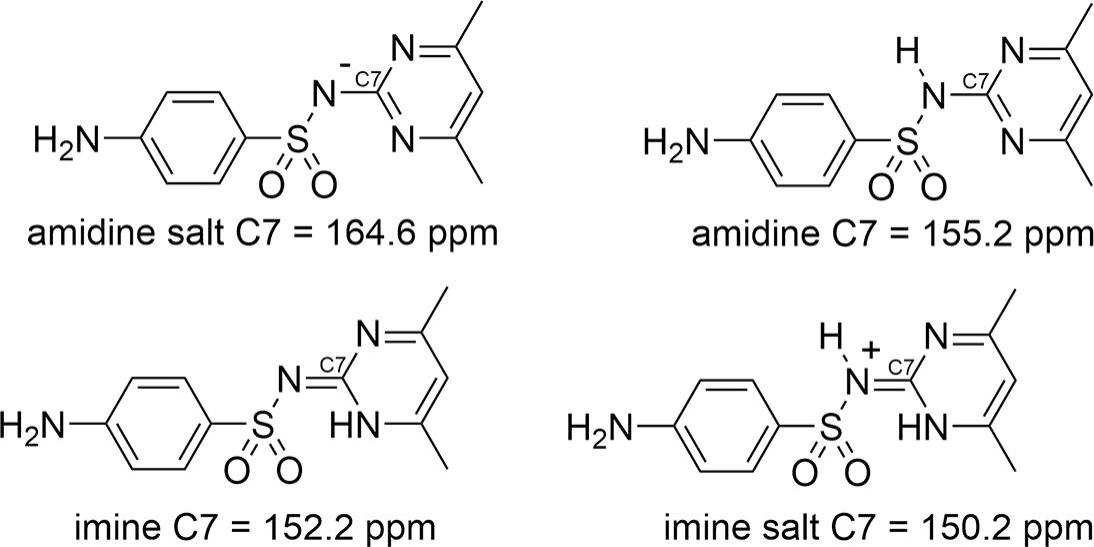
Chemical shift of the C7 ^13^C NMR signal in the different forms of SUL.

The polycrystalline powder of **I** was characterized by ^13^C CP/MAS NMR spectroscopy and each signal represents a chemically different C atom. The ^13^C NMR spectrum of SUL was assigned according to Fu *et al.* (2016 ▸) and Grossjohann *et al.* (2015 ▸), and was used to assign the spectrum of the polycrystalline powder of **I**. The ^13^C NMR spectrum of **I** contained the signals for both SUL and PPD, and most of the ^13^C NMR signals were shifted with respect to the ^13^C NMR spectrum of SUL due to the change in the chemical environment as a consequence of the formation of the salt (Fig. 3 ▸). Evidence of the deprotonation of SUL was observed by the shift to a higher frequency of the C7 signal from 155.9 ppm in SUL to 164.6 ppm in **I** in a similar way to when SUL is deprotonated to form metallic complexes (Hossain *et al.*, 2007 ▸). A similar case is observed when saccharin is deprotonated to form salts with fluoro­quinolones, since the ^13^C NMR signal of the carbonyl C atom (next to the negatively charged N atom) is shifted from 164.0 to 172–173 ppm after deprotonation (Romañuk *et al.*, 2009 ▸). A comparison of the C7 chemical shifts, obtained from solid-state ^13^C NMR spectroscopic analysis reported for co­crystals of SUL in the amidine form, and co­crystals and salts in the imine form (Fig. 4 ▸), revealed that in the amidine form, the C7 (C—NH) signal appeared at 155.2 ppm in the sul­fa­methazine–4-amino­salicylic acid co­crystal (similar to free SUL) (Grossjohann *et al.*, 2015 ▸), while in the sul­fa­methazine–saccharin co­crystal, where SUL adopts the imine form, the C7 (C=N) signal was shifted to a lower frequency, appearing at 152.7 ppm due to shielding caused by the formation of the C=N bond. In the sul­fa­methazinium saccharinate imine salt, the C7 signal (C=NH^+^) appeared at 150.2 ppm (shifted to a lower frequency), showing greater shielding due to the protonation of the C=N bond (Fu *et al.*, 2016 ▸) (Fig. 4 ▸). On the other hand, when amidine SUL is deprotonated as in **I**, the C7 (C—N^−^) signal appears at a higher frequency with respect to free SUL, because it is deshielded as a consequence of the negatively charged nitro­gen effect. Whole assignments of the ^13^C NMR signals are included in Table 2 ▸.

**Figure 5:**
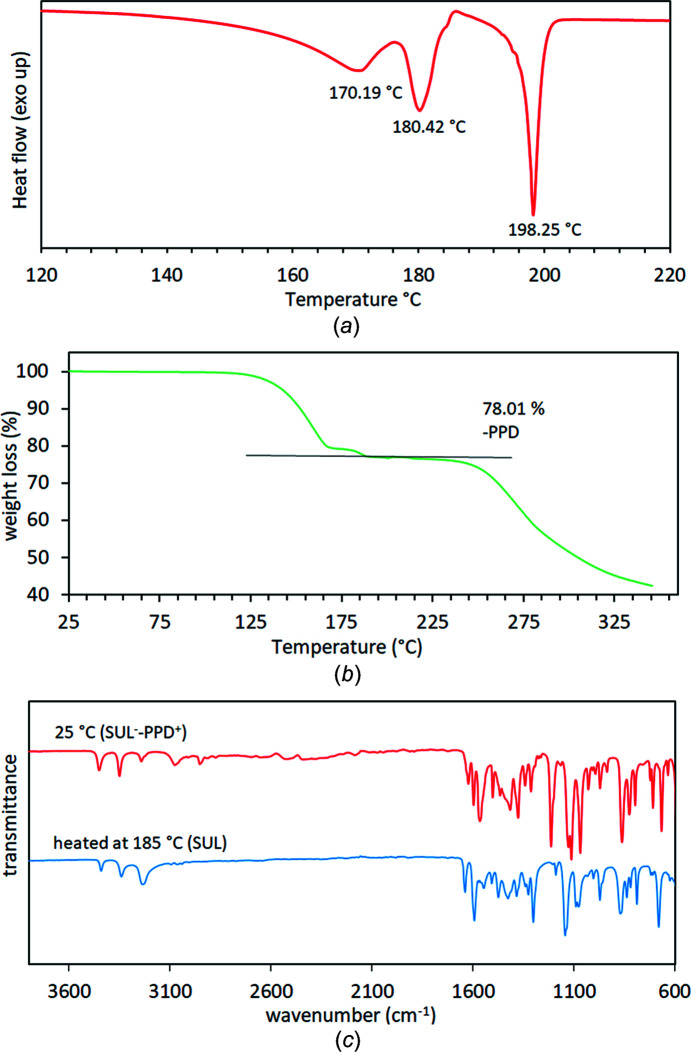
(*a*) The DSC curve of crystal **I**, (*b*) the TGA curve of crystal **I** and (*c*) the IR spectra of the polycrystalline powder of **I** before and after heating.

The thermal properties of the single crystal of **I** were investigated by TGA/DSC. The DSC plot of crystal **I** showed three endothermic peaks (Fig. 5 ▸). Considering that the crystal structure of **I** is composed of only PPD and SUL, the peak at 170.19 °C is assigned to a solid–solid transition before the evaporation of PPD, the peak at 180.42 °C is assigned to the evaporation of PPD and the peak at 198.25 °C is attributed to the melting of SUL (Singh & Baruah, 2019 ▸). The TGA plot showed a weight loss of 21.99% at 185 °C, corresponding to the loss of PPD, as suggested by the DSC curve (Fig. 5 ▸). The mass loss after the melting of SUL is attributed to the degradation of SUL. To confirm the loss of PPD, a 100 mg sample of the polycrystalline powder of **I** was heated at 185 °C for 1 h, then an IR spectrum was recorded and compared with that obtained at room tem­per­ature (25 °C). The IR spectrum of **I** obtained at 185 °C is similar to the IR spectrum of pure SUL, confirming the loss of PPD (Fig. 5 ▸).

### Crystal structure of I

**Figure 6:**
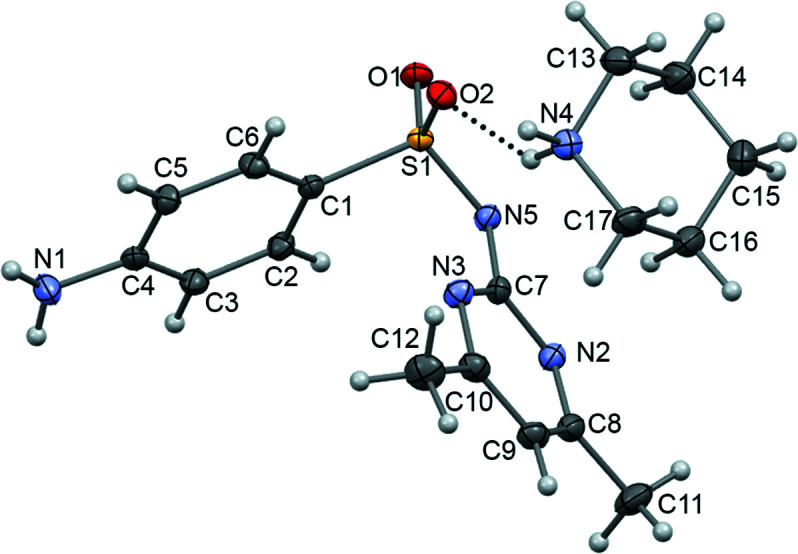
The asymmetric unit of **I**, showing the atom numbering. Displacement ellipsoids are drawn at the 50% probability level. Dashed lines represent hydro­gen bonds.

**Figure 7:**
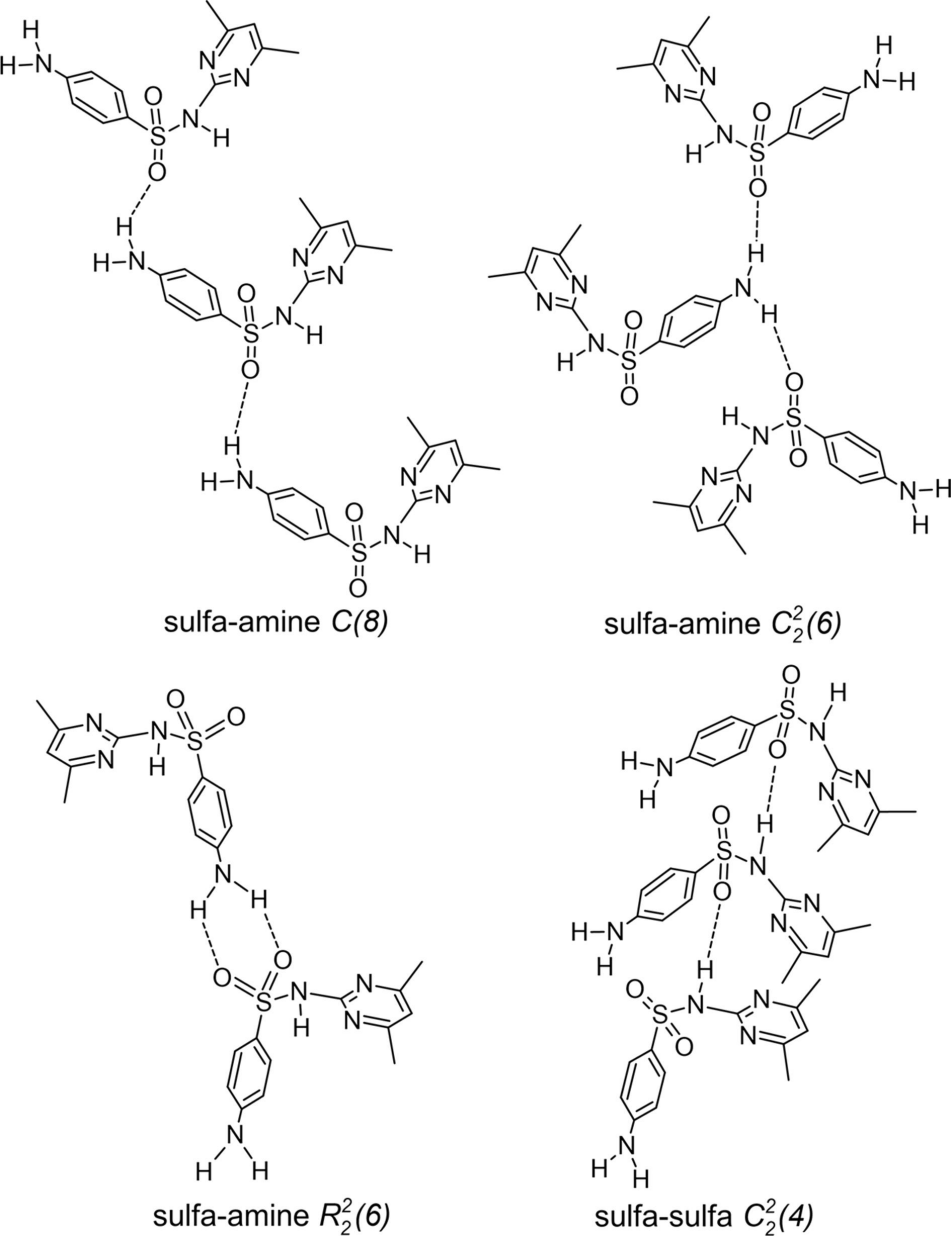
The hydro­gen-bond patterns adopted in the self-assembly of SUL.

**Figure 8:**
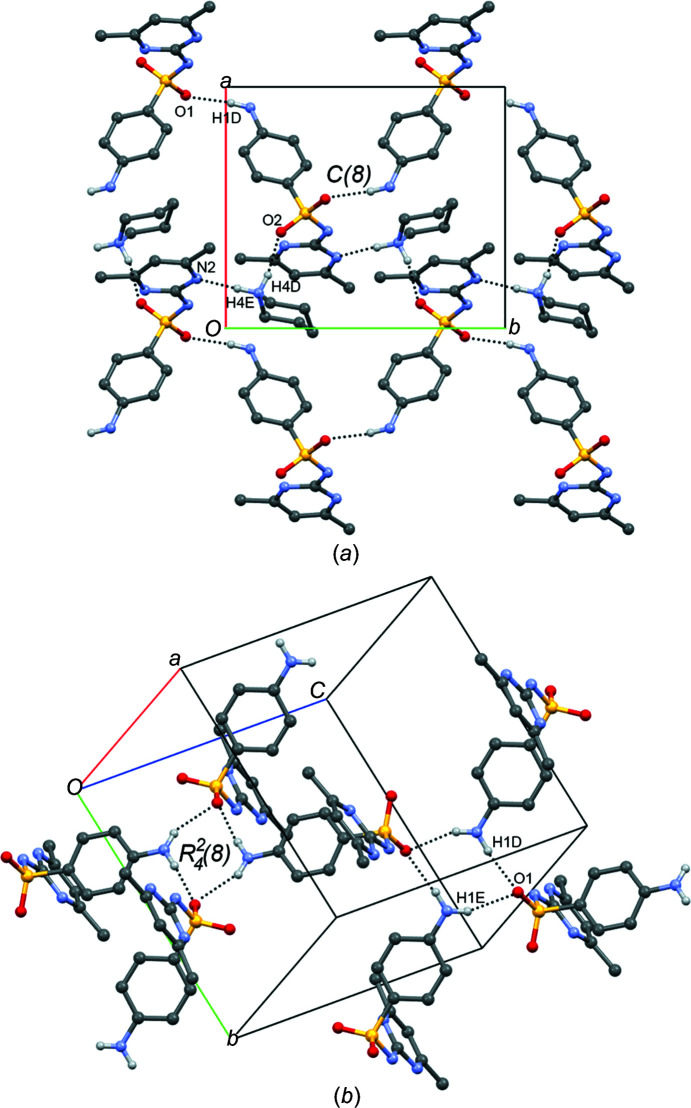
(*a*) The supra­molecular sheet of **I** formed by inter­linked *C*(8) chains of SUL^−^ anions extended along the *ab* plane. (*b*) The inter­connection of SUL^−^ anions depicting the 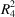 (8) hydro­gen-bond motif. Some H atoms have been omitted for clarity. Dashed lines represent hydro­gen bonds.

Salt **I** crystallized in the space group *P*2_1_/*n* with one SUL^−^ anion and one PPD^+^ cation in the asymmetric unit (*Z* = 4) (Fig. 6 ▸) connected by an N—H^+^⋯O=S (N4—H4*D*⋯O2) hydro­gen bond. The crystal structure of salt **I** showed deprotonation of the sulfonamide N atom of SUL and protonation of the amine group of PPD (to form the pi­peri­dinium group), as predicted by the Δp*K_a_* criteria. The SUL^−^ anion adopts a V shape, with a C1—S1—N5—C7 torsion angle of −61.39 (11)°. A search performed in the the CSD (accessed September 2022; Groom *et al.*, 2016 ▸) for crystal structures of pure SUL and its co­crystals and salts in the amidine form, revealed that in the self-assembly of SUL mol­ecules, four supra­molecular patterns are preferred (three involving amine–sulfa inter­actions and one involving a sulfa–sulfa inter­action) (Fig. 7 ▸). In salt **I**, the SUL^−^ anion adopts the sulfa–amine *C*(8) pattern (Bernstein *et al.*, 1995 ▸) formed by the N1—H1*D*⋯O1^i^ hydro­gen bond, producing a supra­molecular tape running along the *b* axis. Hydrogen-bond details and symmetry codes of crystal **I** is given in Table 3 ▸. The two-dimensional supra­molecular array is formed by the inter­linking of *C*(8) SUL^−^ tapes with the PPD^+^ protons (N4—H4*D*⋯O2 and N4—H4*E*⋯N2^iii^; Table 3 ▸), giving rise to a supra­molecular sheet extended along the *ab* plane (Fig. 8 ▸). Supra­molecular sheets are linked by N1—H1*E*⋯O1^ii^ and N1—H1*D*⋯O1^i^ hydrogen bonds involving two SUL^−^ anions and two PPD^+^ cations, showing an 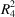 (8) hydro­gen-bond motif in a similar manner to the sul­fa­methazine–fumaric acid co­crystal (Fig. 8 ▸) (Ghosh *et al.*, 2011 ▸).

The crystal structure of pure SUL adopts the sulfa–sulfa 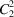 (4) pattern (Fig. 7 ▸) and, after grinding, SUL transfers a proton to PPD (evidenced by the shift of the C7 signal in the ^13^C CP/MAS NMR spectrum and the appearance of the band at 3067 cm^−1^ in the IR spectrum) to form the PPD^+^·SUL^−^ salt, **I**, displaying the sulfa–amine *C*(8) motif, changing the hydro­gen-bonding pattern (evidenced by the shifts in the IR bands) and the chemical environment (shifting the ^13^C NMR signals). Heating **I** at 185 °C leads to the loss of PPD (according to DSC/TGA information and IR spectra) and the remaining SUL returns to the sulfa–sulfa 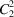 (4) pattern before melting at 198.25 °C.

## Conclusions

The salt pi­peri­dinium sul­fa­methazinate, PPD^+^·SUL^−^, **I**, was obtained by solvent-assisted grinding. Proton transfer was confirmed by IR spectroscopy, solid-state ^13^C NMR spectroscopy and single-crystal X-ray diffraction. The complete transformation of the starting material into the new crystalline phase was confirmed by PXRD analysis. The IR spectra and the PXRD patterns of the polycrystalline powder and the single crystal of **I** matched well, indicating a structural homogeneity between the polycrystalline powder and the single crystal. The crystal structure of salt PPD^+^·SUL^−^ revealed a 1:1 stoichiometry and the SUL^−^ anion adopts the sulfa–amine *C*(8) hydro­gen-bond pattern, forming two-dimensional supra­molecular sheets. Thermal analysis showed the loss of PPD before the melting of SUL.

## Supplementary Material

Crystal structure: contains datablock(s) I, global. DOI: 10.1107/S2053229622012050/vp3027sup1.cif

Structure factors: contains datablock(s) I. DOI: 10.1107/S2053229622012050/vp3027Isup2.hkl

CCDC reference: 2214090

## Figures and Tables

**Table 1:** Experimental details

Crystal data	
Chemical formula	C_5_H_12_N^+^·C_12_H_13_N_4_O_2_S^−^
*M*_r_	363.48
Crystal system, space group	Monoclinic, *P*2_1_/*n*
Tem­per­ature (K)	130
*a*, *b*, *c* (Å)	10.5713 (7), 12.1313 (8), 14.3623 (10)
β (°)	97.182 (7)
*V* (Å^3^)	1827.4 (2)
*Z*	4
Radiation type	Mo *K*α
μ (mm^−1^)	0.20
Crystal size (mm)	0.53 × 0.43 × 0.34

Data collection	
Diffractometer	Agilent Xcalibur Atlas Gemini
Absorption correction	Analytical (*CrysAlis PRO*; Agilent, 2013 ▸)
*T*_min_, *T*_max_	0.93, 0.945
No. of measured, independent and observed [*I* > 2σ(*I*)] reflections	10086, 4298, 3667
*R*_int_	0.025

Refinement	
*R*[*F* ^2^ > 2σ(*F* ^2^)], *wR*(*F* ^2^), *S*	0.037, 0.102, 1.04
No. of reflections	4298
No. of parameters	241
H-atom treatment	H atoms treated by a mixture of independent and constrained refinement
Δρ_max_, Δρ_min_ (e Å^−3^)	0.37, −0.44

Computer programs: *CrysAlis PRO* (Agilent, 2013 ▸), *SHELXT2018* (Sheldrick, 2015*a* ▸), *SHELXL2018* (Sheldrick, 2015*b* ▸) and *ORTEP* for Windows (Farrugia, 2012 ▸).

**Table 2:** CP/MAS solid-state ^13^C NMR chemical shifts (ppm) of SUL and **I**

	SUL	**I**		SUL	**I**
C1	126.1	126.1	C9	117.9	114.0
C2	130.8	132.6	C10	167.0	166.2
C3	114.4	111.2	C11	22.0	24.3
C4	153.9	151.1	C12	21.1	23.6
C5	115.8	111.2	C_a_	–	21.9
C6	130.8	129.9	C_b_	–	21.9
C7	155.9	164.6	C_c_	–	46.6
C8	169.9	169.8			

**Table 3:** Hydrogen-bond geometry (Å, °)

*D*—H⋯*A*	*D*—H	H⋯*A*	*D*⋯*A*	*D*—H⋯*A*
N1—H1*D*⋯O1^i^	0.88 (2)	2.06 (2)	2.9383 (18)	175.1 (17)
N1—H1*E*⋯O1^ii^	0.835 (19)	2.251 (19)	3.0397 (17)	157.6 (18)
N4—H4*D*⋯O2	0.893 (18)	2.157 (18)	2.8980 (16)	140.0 (15)
N4—H4*E*⋯N2^iii^	0.951 (19)	1.865 (19)	2.8085 (18)	171.0 (15)

Symmetry codes: (i) 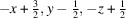 ; (ii) 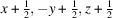 ; (iii) 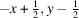 , 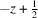 .
